# Double Biocatalysis Signal Amplification Glucose Biosensor Based on Porous Graphene

**DOI:** 10.3390/ma10101139

**Published:** 2017-09-27

**Authors:** Yaping He, Jianbin Zheng, Bini Wang, Hongjiang Ren

**Affiliations:** 1 School of Chemical Engineering, Xi’an University, Xi’an 710065, Shaanxi, China; hjren@xawl.edu.cn; 2 Institute of Analytical Science/Shaanxi Provincial Key Laboratory of Electroanalytical Chemistry, Northwest University, Xi’an 710069, Shaanxi, China; 3 College of Food Engineering and Nutritional Science, Shaanxi Normal University, No. 620, West Chang’an Avenue, Chang’an District, Xi’an 710119, Shaanxi, China; biniwang@snnu.edu.cn

**Keywords:** biological amplification, glucose oxidase, horseradish peroxidase, porous graphene, Au nanoparticles

## Abstract

Controllable preparation of nanopores to promote the performance of electrochemical biosensing interfaces has become one of the researching frontiers in biosensing. A double biocatalysis signal amplification of glucose biosensor for the study of electrochemical behaviors of glucose oxidase (GOx) was proposed by using horseradish peroxidase biosynthesized porous graphene (PGR) as the platform for the biocatalytic deposition of gold nanoparticles (AuNPs). The biosensor showed a linear range from 0.25 to 27.5 μM with a detection limit of 0.05 μM (S/N = 3) towards glucose. Furthermore, the proposed AuNPs/GOx–PGR modified glassy carbon electrode (AuNPs/GOx–PGR/GCE) achieved direct electron transfer of GOx.

## 1. Introduction

Nanomaterials can be used to improve the catalytic performance, impart higher sensitivity, and enhance selectivity to an electrochemical measurement of analysts [1]. Different scales endow nanomaterials with regulable selectivity and sensitivity towards the electrochemical performance of crotonaldehyde, glucose, and electric methanol [2,3,4]. Especially, porous nanomaterials with loose structure can provide more reaction location for electrochemical reaction [5], endowing a better electrochemical response. It can react with atoms, ions, molecules, and nanoparticles on the surface and interior [6]. Additionally, the large surface area make it can load a lot of electroactive material and increase electron transfer rate. The existence of pore structure reduces the diffusion resistance of the electrolyte, which paves a smooth channel for the transference of ions and electrons [7]. Further, electrodes modified with porous nanomaterials change the distribution of the electric double layer on the interface. Effective performance improvement of electrochemical biosensing can be achieved by adjusting the pore structure and properties of porous nanomaterials [8]. Dergunov et al. controlled the particle behavior in nanopores by adjusting the pH value [9]. The investigation of electrochemical sensing behavior on porous nanogold surface indicated that [10] the special structure effectively improves the electrode direct electronic window, eliminate pH interferences, and promote biological activity. Proof–of–principle studies of DNA sequencing have been realized by a combination of engineered biological nanopores with polymer-based positional control [11]. Obviously, employment of nanopore structure in electrochemical sensing can improve its performance. Synthetic nanopores have tunable properties such as size, geometry, and surface chemistry, as well as enhanced mechanical, thermal, and chemical stability play an important role in the fabrication of novel sensing interface tunable property.

The good stability of carbon nanomaterials (CNMs) make themwidely applied to fabricate biosensors for electroanalytical investigations [12,13,14]. The relatively wide potential window, low background current, high catalytic properties, highly loading of biocatalysts, unique structure, and excellent electrical conductivity [15,16] are beneficial for electrochemical processes. Graphene (GR) is a novel two–dimensional layered CNM with high specific surface area, preferable operability, and excellent electrical performance, and has been widely used in the fabrication of high–performance electrochemical sensors [17]. If some carbon atoms are removed from GR [18], a structure forms that generates pores with the edge of unsaturated carbon atoms, named as porous graphene (PGR). The PGR with specific structure tends to capture atoms and ions, forming a saturated bond. Furthermore, PGR can provide an active site for selective adsorption. Based on PGR, an electrochemical DNA migration sensing platform has been fabricated by researchers [19,20]. The results indicated that sensing performance could be adjusted by controlling the pore properties. Glucose oxidase (GOx) has been immobilized on PGR to realize direct electrochemistry and electrocatalysis with the significant advantages of sensitivity and linear range [21]. At present, electronic breakdown, microwave puffing technology, Pickering microemulsion method, and polystyrene template method [22,23,24] have been employed to prepare PGR. Pore structure on GR plane was generated by the internal oxidation induced by the catalysis of horseradish peroxidase (HRP) towards horseradish peroxidase [25]. The oxygen-containing groups on GR tend to close the heme center of HRP and other metal catalytic center enzymes, leading to a greater loading amount of the sensitive sensing film, which favors the selectivity and sensitivity of sensing. Compared with current carbon material modification methods for catalytic reaction [26,27,28], the modification of carbon nanomaterial by biocatalysis can be completed under moderate conditions, without heating, ultrasonicating, or strong acid or basic solutions. Moreover, the final nanocomplex by biocatalysis could have better catalytic ability for biosensing, due to the intrinsic interaction between enzymes and nanomaterials and accelerate electron transfer rate.

The aim of the present work is to controllably prepare PGR with versatile pore size by HRP-induced process, and then conduct in situ biocatalytic deposition of gold nanoparticles (AuNPs) onto the GOx–embedded PGR film to fabricate a novel biosensor for the determination of glucose level with the growth reaction. PGR is prepared by biocatalysis process of HRP and in situ biocatalytic deposition of AuNPs onto PGR by GOx. The two biocatalysts can play the role of double signal amplification to improve the sensitivity of the glucose biosensor. Herein, PGR can act not only as an enzyme immobilization reagent, but also as the biosensing platform for the determination of glucose level. Furthermore, the electrochemical behaviors of GOx at the proposed AuNPs/GOx–PGR/GCE (glassy carbon electrode) are also studied. It is expected that the proposed method has the advantage of design compatibility [29,30,31,32], which gives the electrochemical sensors the potential to be integrated for other devices and electronics.

## 2. Experimental

### 2.1. Reagents

GOx (E.C. 1.1.3.4, 182 Umg^−1^, Type X–S from Aspergillusniger). Chloroauric acid tetrahydrate (HAuCl_4_·4H_2_O) and HRP were purchased from Sigma (Los Angeles, CA, USA). High–purity graphite powder was purchased from the Shanghai Carbon Plant (Shanghai, China). Glucose stock solution was allowed to mutarotate at room temperature overnight before use. All other chemicals were of analytical reagent grade, and doubly distilled water was used in all the experiments. A 0.1 M pH 7.0 sodium phosphate buffered saline (PBS) solution was used in all electrochemical studies unless otherwise stated.

### 2.2. Apparatus and Measurements

A Jeol Neoscope Benchtop scanning electron microscope (SEM) was used for SEM images (Japan Electron Company, Tokyo, Japan). Transmission electron microscope (TEM) images were collected on an E.M. 912 Ω energy–filtering TEM (120 kV) (Japan Electron Company, Tokyo, Japan). A DL–180 ultrasonic cleaning machine (35 KHz, Zhejiang Haitian Electron Instrument Factory, Kunshan, China) was used to dissolve and form a homogeneous solution. All electrochemical experiments were carried out on a CHI660e electrochemical workstation (Shanghai CH Instrument Co. Ltd., Shanghai, China) using a three electrode system. The working electrode was a GCE or a modified GCE. A saturated calomel electrode (SCE) and a platinum electrode served as reference and counter electrodes, respectively. All the electrochemical experiments were conducted at room temperature (25 ± 2 °C).

### 2.3. Biosynthesized Porous Graphene 

First, 80 μL of 5 mg/mL of HRP was added into 1 mg/mL, 2 mg/mL, 4 mg/mL, and 6 mg/mL graphene oxide (GO) solutions and shaken in a rocking incubator at 37 ± 0.2 °C. Then, 20 μL 3 M H_2_O_2_ was added into the above solution, a 24 h interval passed, the addition of H_2_O_2_ was repeated and the operation was last for several days. The product was freeze-dried and stored in the refrigerator (4 °C) for later use.

### 2.4. Preparation of GOx-Modified Electrode

Before use, a glassy carbon electrode (GCE) of 3 mm diameter was polished to a mirror-like finish with 1.0, 0.3, and 0.05 μm Al_2_O_3_ slurry on a polish cloth and rinsed with double–distilled water, then sonicated in ethanol and double-distilled water for 5 min, respectively.

GOx–PGR composite was prepared by dispersing 1 mg PGR and 5 mg GOx into 1 mL PBS by shaking in a rocking incubator at 37 ± 0.2 °C, mixing for 24 h, then casting 10 μL of the mixture onto the surface of the bare GCE by using a syringe to prepare GOx–PGR/GCE. Without use, the biosensor was stored at 4 °C in a refrigerator.

### 2.5. Growth of AuNPs

All growth solutions were purged with highly purified oxygen for 30 min before experiments, and an oxygen environment was kept over the solution during the growth process. The GOx–PGR/GCE was immersed into a growth solution consisting of 0.2 mM HAuCl_4_ in 0.1 M PBS with different concentrations of glucose for 25 min, then it was removed from the above solution and transferred into 0.1 M PBS.

## 3. Results and Discussion

### 3.1. The Controllable Biosynthesized Porous Graphene

**Figure 1:**
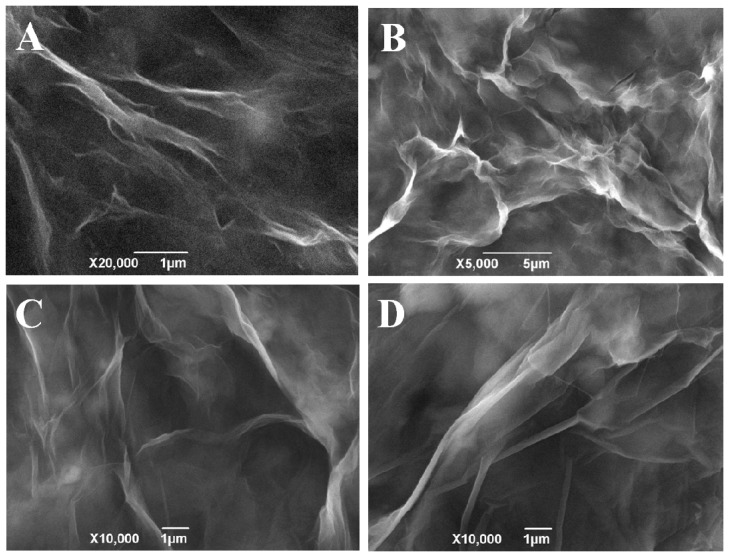
SEM images of product with different reaction concentrations of graphene oxide (GO) for 15 days: (**A**) 1 mg/mL; (**B**) 2 mg/mL; (**C**) 4 mg/mL and (**D**) 6 mg/mL.

**Figure 2:**
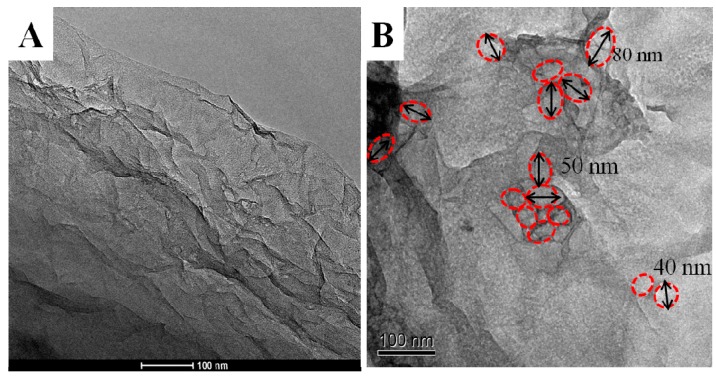
TEM images of product with reaction concentrations of GO 2 mg/mL for 15 days with low (**A**) and high order (**B**).

The pore structure on PGR was adjusted by reaction concentrations of GO and biocatalytic induction time. By comparison, 15 days was the optimal reaction time. At the beginning, there were only a few pores on GR. As the reaction time increased, the number of pore substantially increased. However, the amount of pores did not change further after 15 days. SEM images of products with different reaction concentrations of GO are shown in Figure 1. When GO concentration was 1 mg/mL, the pore structure was easily formed on GR with limited number. Increasing the concentration to 2 mg/mL, the number of pores substantially increased with uniform pore size. Further increasing of GO concentration did not contribute to the increase of pore number. Further, the pore size was nonuniform, and the size became much larger—on the high order of micrometers. When GO concentration was increased to 6 mg/mL, it was difficult to determine the pore structure on GR. All of the above indicates that the best condition for a biosynthesized pore structure on PGR was 2 mg/mL GO reacted for 15 days. TEM images of the product under optimal conditions are shown in Figure 2. It can be seen that the whole plane shows flake–like shape and nano-thickness GR with remarkable pore structure, as shown in Figure 2A. Pore structures with diameter 40 to 80 nm are labeled with a red line on the GR in Figure 2B. These pores played an important role in the further immobilization of GOx for biocatalytic growth of AuNPs on GR.

### 3.2. The Mechanisms of Biocatalytically–Grown AuNPs

The possible mechanism of the biocatalytic growth of AuNPs has been confirmed by our previous work [33,34]. H_2_O_2_ is produced by the oxidization of glucose from GOx. Then, H_2_O_2_ reduces [AuCl_4_]^−^ into Au (0), catalyzed by HRP on the surface of PGR. A double biocatalysis signal amplification was achieved for the determination of glucose. The sensitivity was greatly improved, with a value 1.2 to 1000 times higher than existing methods.

### 3.3. Optimization of Sensor Performance

**Figure 3:**
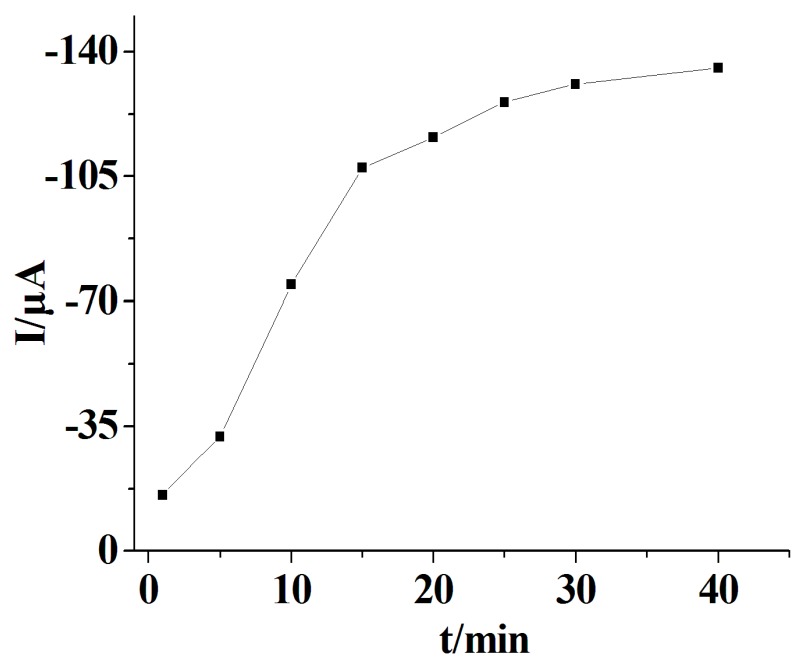
Plots of peak current vs. accumulation time.

The incubation time may affect the growth of AuNPs on the designed GOx–PGR/GCE. Subsequent experiments were carried out to obtain the optimum incubation time for GOx-induced AuNPs formation with a glucose concentration of 100 μM. The dependence of cyclic voltammetry (CV) peak current of the biocatalytic accumulation of AuNPs on accumulation time is displayed in Figure 3. The highest peak current was obtained when the accumulation time was 25 min. As the reaction time increased, there was no significant increase of the peak current. Thus, the optimum accumulation time for the deposition of AuNPs was 25 min. Following experiments were carried out under this condition to get the best electrochemical performances.

### 3.4. The Determination of Glucose Level

**Figure 4:**
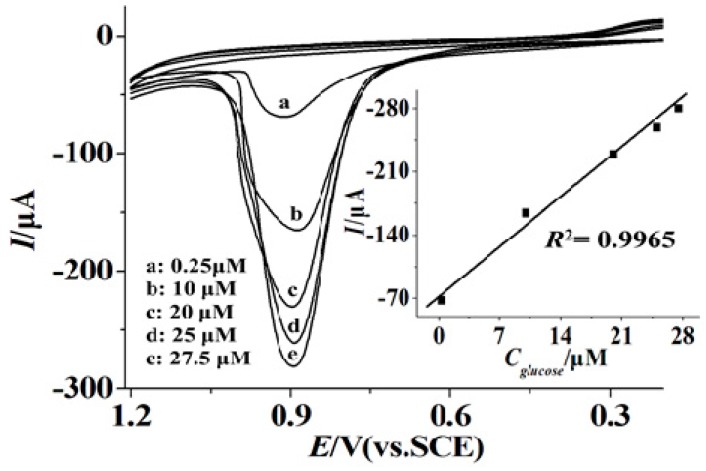
CVs of glucose oxidase (GOx)-induced formation of gold nanoparticles (AuNPs) for increasing concentrations of glucose. Glucose concentration ranges: (**a**) 0.25 μM; (**b**) 10 μM; (**c**) 20 μM; (**d**) 25 μM and (**e**) 27.5 μM. Inset: plot of peak current vs. glucose concentration. Reaction solution: a stirred 0.1 M phosphate buffered saline (PBS) solution containing 0.2 mM HAuCl_4_ and different concentrations of glucose.

Since the current response of AuNPs is related to the concentration of glucose, it can be used for the detection of glucose level. Figure 4 showed CVs on the GOx–PGR/GCE with increasing glucose concentration under the optimum conditions. After a preoxidation process before the detection, the reduction peak at a potential of about 0.4 V is weak due to Au(III), which is not beneficial for the sensitivity improvement. The oxidation peak of Au(III) at the potential of 0.85 V was chosen to describe the determination of glucose level, which was much smaller than our previous work. The current response at the potential of 0.85 V showed a linear relationship with the concentration of glucose. The potential was lower than our previous work [33,34], indicating a better anti-interference ability. The results showed a good linear relationship with the glucose concentration. The linear regression equation was *I_ss_*(μA) = −7.557*C* (μM) − 75.31 (*r* = 0.9945) with linearity from 0.25 to 27.5 μM (inset, Figure 4) and a detection limit of 0.05 μM (S/N = 3). The sensitivity was 755 μA/mM. Compared with other methods, our biosensor showed sensitivity that was higher by 1.2 to 1000 times and a detection limit that was lower by one to three orders of magnitude. According to Michaelis–Menten kinetic mechanism, the apparent Michaelis–Menten constant $K_{M}^{app}$ value was calculated to be 67.4 μM, which was much lower than some previous reports. Herein, PGR offered a promising platform for glucose detection based on the GOx-induced formation of AuNPs. The method might be used to the detect blood sugar of humans in daily life with sweat or tears to intead the necessity of the blood collection of the existing test.

### 3.5. Direct Electrochemistry 

**Figure 5:**
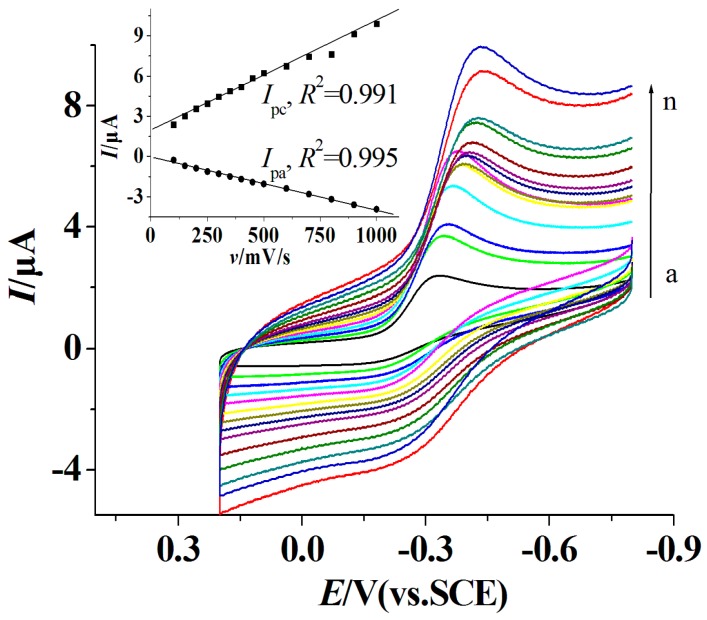
CVs of the AuNPs/GOx–PGR/GCE (glassy carbon electrode) in N_2_ saturated PBS with different scan rates (from a to n: 100, 150, 200, 250, 300, 350, 400, 450, 500, 600, 700, 800, 900, 1000 mV/s).

CVs of the proposed AuNPs/GOx–PGR/GCE with different scan rates are shown in Figure 5. A pair of redox peaks with *E_pa_* = −0.217 V and *E_p_*_c_ = −0.327 V (vs. SCE) are shown with the formal potential *E°′* = −0.272 V, Δ*E_p_* = 90 mV at the scan rate of 100 mV/s. The ratio of cathodic to anodic current intensity is about 1, indicating that a fast direct electron transfer reaction occurred. The value of *E°′* was close to the values reported previously [35,36]. According to the equation *I_p_* = *n*^2^*F*^2^vAΓ*/4*RT* = *nFQv*/4*RT* [37], the surface concentration of electroactive Γ* was calculated to be 2.34 × 10^−12^ mol/cm^2^, which matched the theoretical value (2.86 × 10^−12^ mol/cm^2^) for the monolayer of GOx on the bare electrode surface, indicating that the AuNPs/GOx–PGR/GCE keep the nanostructure of GR.

## 4. Conclusions 

In the present work, HRP–induced PGR with adjustable pore size was employed as the platform for in situ biocatalytic deposition of AuNPs, and a novel double biocatalysis (HRP+GOx) signal amplification glucose biosensor was constructed based on the CV response of AuNPs with different concentrations of glucose. The biosensor showed a low determination limit and high sensitivity for glucose detection. The reason can be ascribed to: (1) The PGR provided a benign microenvironment for the biocatalysis deposition process and the porous structure surface of PGR greatly enlarged the specific surface area to effectively increase the amount of GOx immobilized; (2) The advantages of biocatalysis itself, such as the accumulation and amplification effects; (3) Synergistic effects of PGR and AuNPs not only improved the electron transfer rate of the sensing system, but also exhibited the signal amplification of nanosize materials. Furthermore, the proposed AuNPs/GOx–PGR/GCE achieved the direct electrochemistry of GOx and electrocatalysis towards glucose. The present study may pave the way for the synthesis of novel nanomaterials with unique morphology and properties. Moreover, the advantage of the compatibility of the biosensor design can be integrated for other devices and electronics. Further works employing PGR as the biosensor platform are in our schedule.
